# P3-GemOx as a novel immunochemotherapy candidate in NK/T-cell lymphoma management

**DOI:** 10.3389/fmed.2025.1666601

**Published:** 2025-10-23

**Authors:** Yuyang Zhang, Haiming Kou, Zhipeng Cheng, Xuan Lu, Yu Hu, Liang V. Tang

**Affiliations:** Institute of Hematology, Union Hospital, Tongji Medical College, Huazhong University of Science and Technology, Jiefang Dadao, Wuhan, China

**Keywords:** NK/T-cell lymphoma, immunochemotherapy, prognosis, hematopoietic stem cell transplantation, retrospective studies

## Abstract

**Introduction:**

Natural killer/T-cell lymphoma (NKTL) is a rare disease that shows suboptimal responses to existing therapies. This study reports the efficacy of the P3-GemOx regimen compared to the traditional PP-GemOx regimen in advanced NKTL.

**Methods:**

Eleven patients received the P3-GemOx regimen [mitoxantrone hydrochloride liposome (Plm60) combined with PP-GemOx (anti-PD-1 antibody, pegaspargase, gemcitabine, and oxaliplatin)] every 3–4 weeks, with a median of 3 cycles (range: 1–4). Another eleven patients received the PP-GemOx regimen every 3–4 weeks, with a median of 4 cycles (range: 2–6). Treatment response was assessed using 18F-FDG-PET with CT or MRI.

**Results:**

All patients treated with P3-GemOx responded, with nine achieving complete remission (CR) and two partial remission (PR), yielding an overall response rate (ORR) of 100%. Seven of these patients underwent hematopoietic stem cell transplantation (HSCT). In the PP-GemOx group, the ORR was 63.6%, and no patients underwent HSCT. All adverse events were manageable and resolved.

**Discussion:**

The P3-GemOx regimen demonstrates superior efficacy over the PP-GemOx regimen in advanced NKTL.

## Introduction

NK/T-cell lymphoma (NKTL), a unique entity within peripheral T-cell malignancies, exhibits marked geographic prevalence in East Asia and Latin America (1, 2). Initial clinical presentation predominantly occurs at early disease stages (I-II) in over 65% of cases, with characteristic involvement of upper aerodigestive tract structures (3, 4). Prognostic improvements have been documented through radiation-chemotherapy combinations administered concurrently or sequentially (5–7). However, therapeutic management of advanced-stage disease continues to pose significant challenges, evidenced by 70–80% of patients developing terminal progression within 5 years post-diagnosis (8–11).

Therapeutic protocols for advanced NKTL frequently incorporate L-asparaginase derivatives, including polyethylene glycol-conjugated formulations (12–14). Clinical investigations have validated the P-GemOx protocol as a frontline therapeutic option due to its enhanced therapeutic index. A Chinese multicenter analysis spanning 10 years revealed 71.7% overall response rates with this regimen, accompanied by 33.8% 2-year PFS and 44.5% 2-year OS metrics (15). Notably, primary treatment failure persists in nearly three-quarters of cases, underscoring therapeutic limitations.

Emerging immunochemotherapy approaches show promise, as evidenced by a nine-patient cohort study where PD-1 blockade combined with P-GemOx (PP-GemOx protocol) yielded 88.9% ORR and 77.8% CR rates (16). The results from Tian et al.’s study demonstrated that the PP-GemOx regimen achieved an 85% CR rate and a 100% ORR in the enrolled 34 patients, with a 64% 24-month PFS rate and a 76% 36-month OS rate (17). Despite these advances, the overall response rate with PP-GemOx still has considerable room for improvement, and a subset of patients remains insensitive to this regimen. These limitations highlight the need for innovative pharmacological interventions and optimized therapeutic sequencing to further enhance efficacy and overcome insensitivity.

Within conventional chemotherapeutic arsenals, anthracycline derivatives maintain clinical prominence. The synthetic anthraquinone mitoxantrone mediates cytotoxic effects through DNA intercalation, RNA synthesis disruption, and topoisomerase II inhibition (18). This agent has demonstrated therapeutic utility across multiple malignancies including breast carcinoma (19), prostatic neoplasms (20), lymphoproliferative disorders (21), acute leukemias (22), and neuroinflammatory conditions (23). Recent mechanistic insights reveal mitoxantrone’s capacity to mobilize NK cells and CD8 + CTLs, potentiating granzyme-perforin mediated tumor lysis. Paradoxically, this immunomodulation induces IFN-*γ* mediated PD-L1 upregulation and Treg expansion, potentially counteracting immunogenic cell death mechanisms (24). These dual effects suggest therapeutic synergy when combined with PP-GemOx immunochemotherapy, potentially addressing the limitations of PP-GemOx by enhancing response rates and targeting insensitive populations.

Clinical application of mitoxantrone remains constrained by dose-dependent cardiotoxicity, with extensive literature documenting cardiomyopathy risks (25–27). Safety concerns regarding myocardial dysfunction and secondary leukemogenesis have resulted in stringent usage guidelines, particularly in anthracycline-pretreated populations (18, 28).

Liposomal drug delivery systems offer pharmacological advantages through improved biodistribution profiles, controlled release kinetics, and tumor-selective accumulation (18, 29). Preclinical models demonstrate that pegylated liposomal encapsulation modifies mitoxantrone’s pharmacokinetic profile, enhancing therapeutic efficacy while mitigating systemic toxicity (30). Clinical comparisons reveal superior safety outcomes for pegylated liposomal mitoxantrone versus conventional formulations at equivalent dosages (31).

Capitalizing on mitoxantrone’s immunomodulatory potential and the need to improve upon the PP-GemOx regimen, we developed an innovative therapeutic paradigm combining liposomal mitoxantrone (Plm60) (30, 31) with PP-GemOx for advanced NKTL management. This communication details the clinical efficacy, safety parameters, and exploratory biomarker findings associated with this combinatorial approach, which aims to achieve higher response rates and benefit patients who are insensitive to PP-GemOx.

## Materials and methods

### Patients and treatment

**Figure 1:**
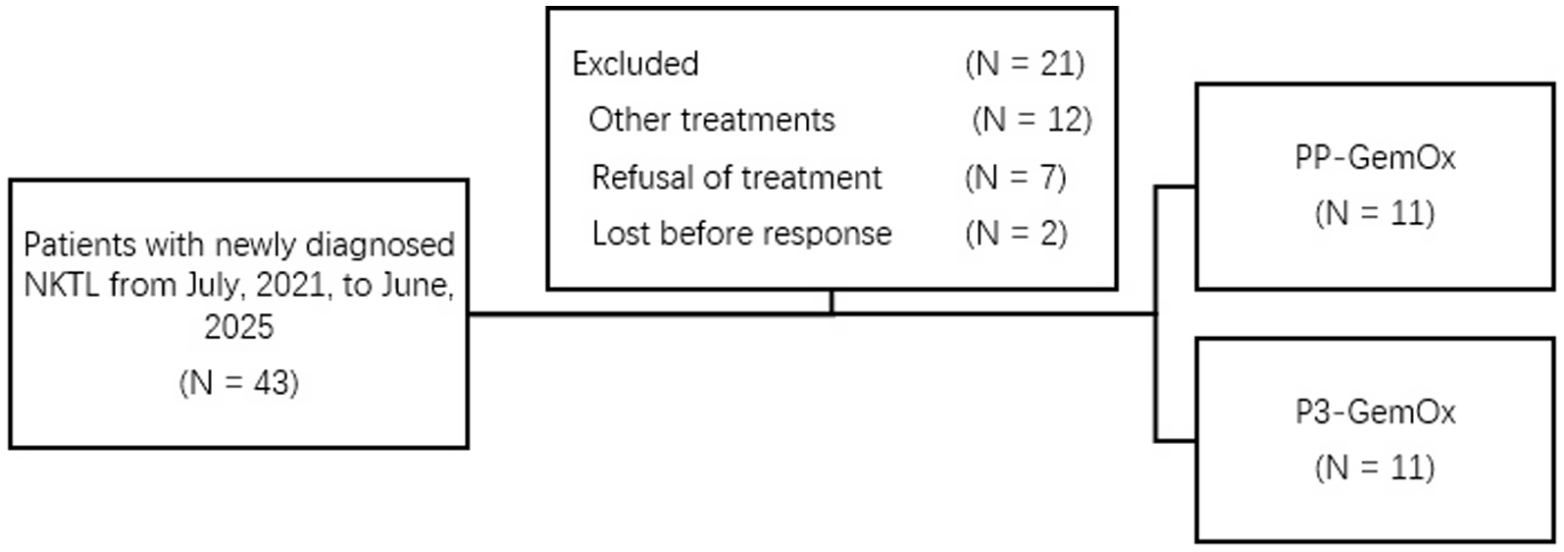
Treatment allocation and number of patients included in the analysis. Other treatments, patients with newly diagnosed NKTL received treatment like SMILE, etc.

Between July 2021 and June 2025, 22 newly diagnosed NK/T-cell lymphoma patients were enrolled at Wuhan Union Hospital (Wuhan, China) (Figure 1). The clinical characteristics of these patients are presented in Supplementary material. Retrospective classification divided these patients into two cohorts: the PP-GemOx group (*n* = 11) and P3-GemOx group (*n* = 11). As a retrospective study, our patients were not originally randomized to receive the different treatment regimens. This is attributable to the fact that PLM60 is a novel agent, and our institution pioneered the P3-GemOx regimen based on PP-GemOx only in recent years. Consequently, the majority of earlier patients received the PP-GemOx protocol, while some clinical teams have begun adopting the P3-GemOx regimen in more recent periods. The PP-GemOx cohort received a median of 4 treatment cycles (range: 2–6), administered every 3–4 weeks and consisting of: Anti-PD-1 antibody (Day 1), Pegaspargase 2000 U/m^2^ (Day 1), Gemcitabine 1 g/m^2^ (Days 1 & 8), Oxaliplatin 130 mg/m^2^ (Day 1). Conversely, the P3-GemOx group underwent a median of 3 cycles (range: 1–4) with the following regimen every 3–4 weeks: Anti-PD-1 antibody (Day 1), Pegaspargase 2000 U/m^2^ (Day 1), Gemcitabine 1 g/m^2^ (Day 1), Oxaliplatin 130 mg/m^2^ (Day 1), Plm60 20 mg/m^2^ (Day 8).

For advanced-stage NK/T-cell lymphoma (NKTL) patients, hematopoietic stem cell transplantation was recommended post-achievement of complete remission (CR) or partial remission (PR) following chemotherapy, contingent upon adequate performance status. All participants received standardized supportive care including anti-infective agents, hematopoietic growth factors, fibrinogen, or platelet transfusions as clinically indicated, while chronic HBV carriers maintained continuous antiviral prophylaxis. During the screening process, we exclusively enrolled newly diagnosed NKTL patients who presented to our hospital, while excluding those with relapsed disease or secondary malignancies. However, patients who had received other treatment regimens as first-line therapy were not excluded.

### Response assessment and monitoring

Treatment response was evaluated using fluorodeoxyglucose positron emission tomography/computed tomography (FDG PET/CT), with assessment based on the standardized 5-point Deauville criteria (32). Additionally, scheduled computed tomography (CT), superficial lymph node ultrasounds, and bone marrow biopsies were performed as needed. Due to the retrospective nature of the study, the assessment schedule varied; however, early evaluations after two cycles were generally adopted (33). Among the cases included in our study, two patients (Cases 5 and 7) did not undergo PET-CT scans during their early post-treatment evaluation after two chemotherapy cycles, likely due to personal financial considerations, and instead received alternative assessments using CT and ultrasound imaging. Additionally, one patient (Case 11) was evaluated only after completing three chemotherapy cycles due to issues with compliance. For patients exhibiting symptoms or signs indicative of central nervous system tumor involvement, cranial MRI scans were conducted, and lumbar punctures were performed when necessary. Circulating EBV DNA levels were quantified using quantitative polymerase chain reaction (34), which was performed on an Applied Biosystems™ 7,500 Real-Time PCR System, with a defined lower limit of detection of 1.00E+01 copies/ml. Complete remission (CR) required confirmation via PET-CT showing no disease, negative bone marrow findings, and undetectable serum EBV DNA. Partial response (PR) was characterized by a ≥50% reduction in the sum of the products of diameters (SPDs) for measurable lesions, without the emergence of new lesions. Patients classified as stable disease (SD) did not meet criteria for CR or PR but also did not satisfy the requirements for progressive disease (PD), defined as either new lesion appearance or a ≥50% increase in SPD from the lowest recorded measurement (nadir). Adverse events (AEs) were categorized and graded according to the National Cancer Institute Common Terminology Criteria for Adverse Events, version 5.0 (NCI CTCAE v5.0).

### Statistical analysis

In this study, comparisons of categorical variables—including patient baseline characteristics, response rates, and incidence of adverse events—were performed using the Chi-square test or Fisher’s exact test, as appropriate, in GraphPad Prism software (version 8.0.2). Survival curve plotting and analysis were also conducted using GraphPad Prism (version 8.0.2), while the landmark analysis was carried out with SPSS software (version 21). Statistical significance was set at *p* < 0.05.

## Results

### Patients

**Figure 2:**
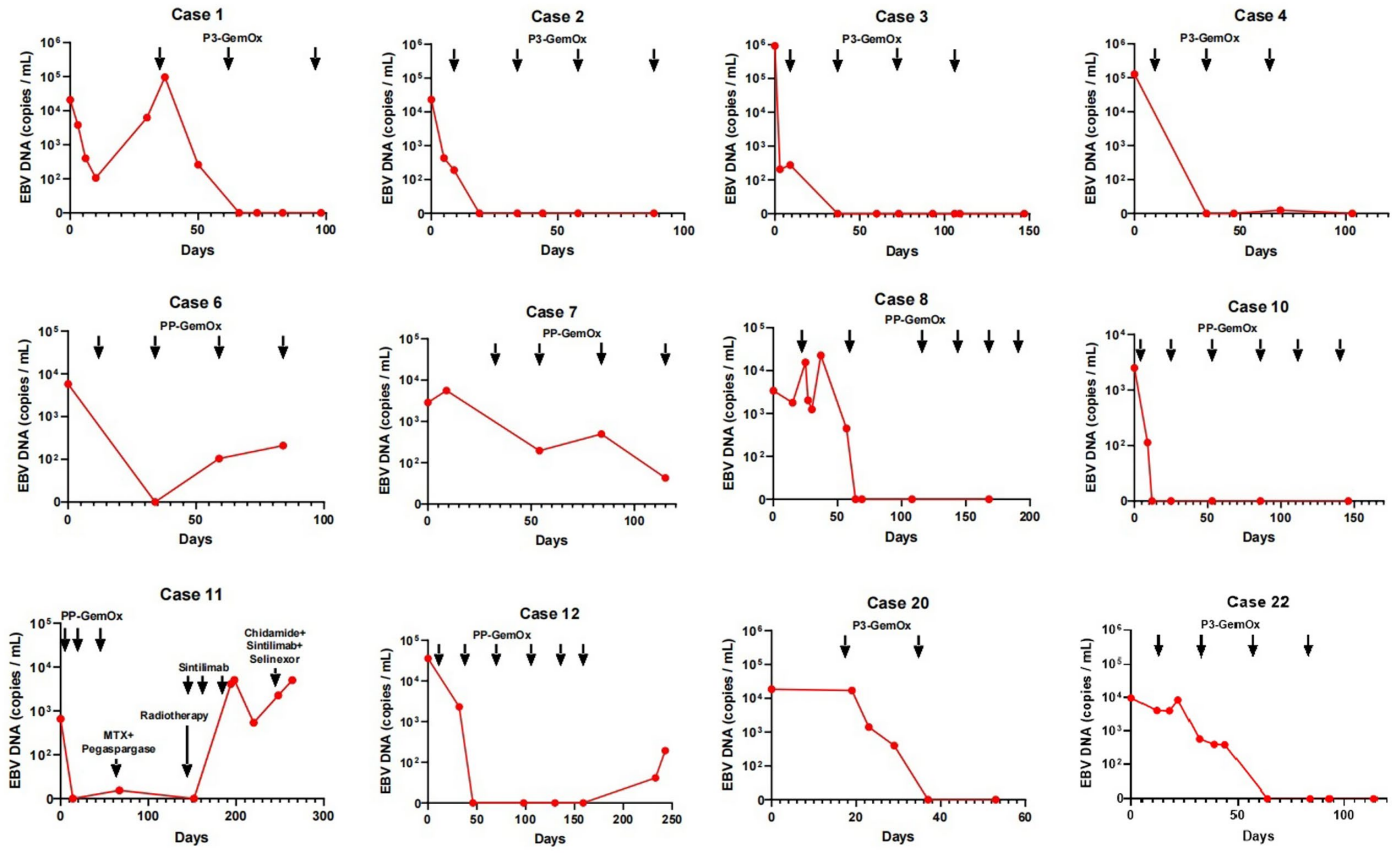
Changes in circulating EBV DNA during therapy. The EBV DNA in cases 1, 2, 3, 4, 20, 22 became normal after 1 or two cycles of P3-GemOx therapy. The EBV DNA in cases 8 and 10 became normal after two cycles of PP-GemOx therapy. The EBV DNA in cases 6 and 7 decreased after one cycle of PP-GemOx therapy, but remains a low level about 1 × 10^2^ copies/mL after four cycles of the therapy. The EBV DNA in case 12 became normal after two cycles of PP-GemOx therapy, but then increased to 1.94 × 10^2^ copies/mL because of treatment interruption. The EBV DNA in case 11 became normal after 2 cycles of PP-GemOx therapy. However, his tumor progressed, EBV levels increased, and despite changing the treatment regimen, it remained uncontrolled.

A total of 22 patients with newly diagnosed NKTL were treated and included in this study. Their median age was 40 (range, 14–76) years. Most had advanced-stage disease with widespread organ involvement (P3-GemOx: I, *n* = 2; IV, *n* = 9; PP-GemOx: I_E_, *n* = 1; II, *n* = 1; II_E_, *n* = 1; IV, *n* = 8; Table 1). Circulating EBV DNA ranged from 660 to 923,000 copies/mL (Figure 2). The patients’ other characteristics are summarized in Tables 1, 2.

**Table 1:** Patient demographic and clinical characteristics.

Characteristic	No. of patients (%) in PP-GemOx group	No. of patients (%) in P3-GemOx group	*p*-value
Age			>0.99
≤60	10 (90.9%)	11 (100%)	
>60	1 (9.1%)	0 (0%)	
Sex			>0.99
Men	7 (63.6%)	7 (63.6%)	
Women	4 (36.4%)	4 (36.4%)	
ECOG performance status			0.22
0	0 (0%)	1 (9.1%)	
1	9 (81.8%)	10 (90.9%)	
2	2 (18.2%)	0 (0%)	
Ann Arbor stage			0.28
I	0 (0%)	2 (18.2%)	
I_E_	1 (9.1%)	0 (0%)	
II	1 (9.1%)	0 (0%)	
II_E_	1 (9.1%)	0 (0%)	
IV	8 (72.7%)	9 (81.8%)	
‘B’ symptom			0.12
Absent	4 (36.4%)	1 (9.1%)	
Present	7 (63.6%)	10 (90.9%)	
Serum LDH			0.27
Normal	3 (27.3%)	1 (9.1%)	
Increased	8 (72.7%)	10 (90.9%)	
PINK score			>0.99
0–2	3 (27.3%)	3 (27.3%)	
3–4	8 (72.7%)	8 (72.7%)	
Bone marrow invasion			0.39
Absent	6 (54.5%)	4 (36.4%)	
Present	5 (45.5%)	7 (63.6%)	
EBV DNA load			0.17
Low (<4 × 10^2^ copies/ml)	5 (45.5%)	2 (18.2%)	
High (≥4 × 10^2^ copies/ml)	6 (54.5%)	9 (81.8%)	

ECOG, Eastern Cooperative Oncology Group. Differences between groups (*p*-value) were calculated by chi-square test.

**Table 2:** Therapies and outcomes of 20 patients with NKTL treated with the immunochemotherapy regimen.

Case	CTx (cycles)	Outcome (cycles)	Other Rx	Survival, mo
1	P3-GemOx (3)	CR (3)	Allo-HSCT	24+
2	P3-GemOx (4)	CR (4)	N/A	22+
3	P3-GemOx (4)	CR (4)	Allo-HSCT	20+
4	P3-GemOx (3)	CR (3)	Allo-HSCT	26+
5	COEPL (2)	SD (2)	N/A	–
	P3-GemOx (4)	PR (3)		21+
6	PP-GemOx (4)	PR (3)	N/A	18+
7	PP-GemOx+chidamide (4)	PD (4)	N/A	7 LTF
8	PP-GemOx (6)	PR (4)	N/A	20 LTF
9	PP-GemOx (1)	PD (1)	N/A	–
	P3-GemOx (1)	CR (1)		25+
10	PP-GemOx (6)	PR (4); PD (6), DOD	N/A	9
11	PP-GemOx (3)	PD (3), DOD	Radiotherapy	10
12	PP-GemOx (6)	PR (4); CR (6)	N/A	25+
13	PP-GemOx (2)	PR (2)	N/A	25+
14	P3-GemOx (3)	PR (3)	Allo-HSCT	12.8+
15	PP-GemOx+chidamide (5)	PR (2); CR (5)	N/A	13.2+
16	PP-GemOx (1)		Splenectomy	–
	P3-GemOx (3)	CR (3)	Allo-HSCT	13.7+
17	PP-GemOx (4)	DOD	N/A	5
18	PP-GemOx (4)	PR (3)	N/A	12.5+
19	PP-GemOx (2)	DOD	N/A	3
20	P3-GemOx (2)	CR (2)	Allo-HSCT	14+
21	PP-GemOx (1)	SD (1)		
	P3-GemOx (2)	CR (2)	N/A	12+
22	P3-GemOx (4)	CR (2)	Allo-HSCT	13+

Allo-HSCT, allogeneic hematopoietic stem-cell transplantation; CR, complete response; PR, partial response; SD, stable disease; PD, progressive disease; CTx, chemotherapy; COEPL, cyclophosphamide, etoposide, vincristine, prednisone, and pegaspargase; LTF, loss-to-follow-up; DOD, dead of disease.

### Response to the PP-GemOx regimen

**Figure 3:**
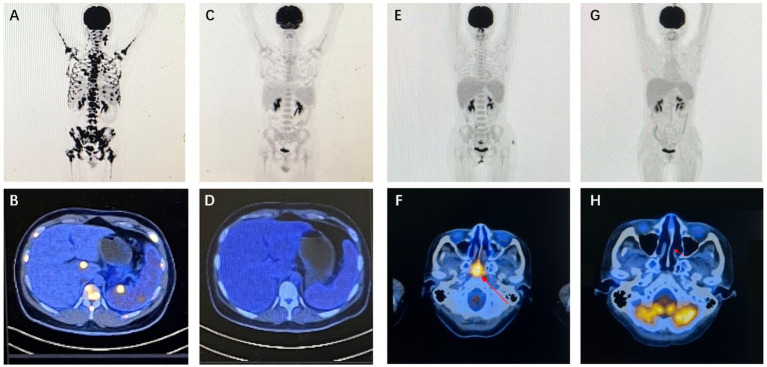
Imaging findings of representative patients. Case 20 PET/CT results: baseline PET/CT demonstrated systemic lymph node involvement, splenomegaly with focally increased metabolic activity, and concurrent intramedullary lesion infiltration **(A,B)**. Following two cycles of P3-GemOx therapy, follow-up PET/CT confirmed complete resolution of all FDG-avid lesions, achieving complete response (CR) **(C,D)**. Case 22 PET/CT results (study obtained after initial chemoradiotherapy): baseline PET/CT revealed soft tissue thickening with intensely increased metabolism in the posterosuperior wall and bilateral walls of the nasopharynx, suggestive of neoplastic lesions **(E,F)**. After two cycles of P3-GemOx therapy, follow-up PET/CT documented complete resolution of all FDG-avid lesions, confirming complete response (CR) **(G,H)**.

A median of four cycles (range, 2–6) of the PP-GemOx regimen were administered. An objective response was observed in seven of the 11 patients, with five achieving a partial response (PR) and two achieving complete response (CR). These seven patients who met the criteria for transplantation declined to undergo hematopoietic stem cell transplantation due to personal reasons. Notably, one patient (case 12) attained PR after four cycles and then achieved CR after six cycles. Another patient (case 15) achieved PR after two cycles, subsequently progressing to CR after five cycles. Additionally, one patient (case 10) reached PR following four cycles, but her condition deteriorated after two months, leading to seizures and eventual death due to respiratory failure from central nervous system infiltration. Another patient (case 7) presented with progressive disease (PD) during the post-chemotherapy assessment and declined further treatment for personal reasons, resulting in loss to follow-up. Patient case 11 also presented with PD during the post-chemotherapy evaluation, accompanied by an increase in Epstein–Barr virus (EBV) DNA. This patient developed a severe lung infection during treatment and ultimately succumbed to septic shock. Patient case 17 experienced severe neutropenia after four cycles and subsequently developed a serious lung infection, leading to death from septic shock. Moreover, patient case 19, whose primary tumor was located in the transverse colon, suffered a sudden colon perforation following two cycles of chemotherapy. The patient declined surgical intervention and ultimately passed away as a result. In this group, both the 1-year progression-free survival (PFS) and overall survival (OS) rates were reported at 54.5% (Figure 3).

### Response to the P3-GemOx regimen

**Figure 4:**
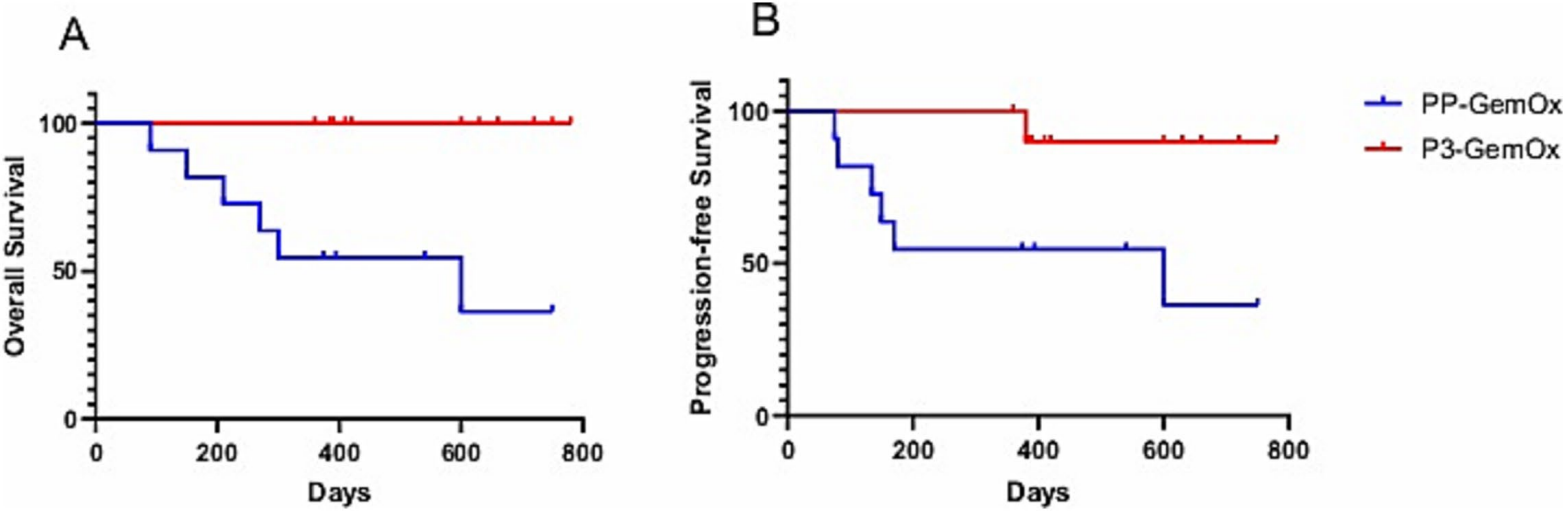
**(A)** Overall survival (OS; PP-GemOx vs. P3-GemOx: *p* < 0.05) and **(B)** progression-free survival (PFS; PP-GemOx vs. P3-GemOx: *p* < 0.05).

A median of three cycles (range, 1–4) of the P3-GemOx regimen were administered, with every patient showing an objective response. Among these, seven of the nine patients achieved complete response (CR), while two attained partial response (PR). Normalization of EBV DNA was observed after one or two cycles of therapy. Seven patients successfully undergoing hematopoietic stem cell transplantation (HSCT), and other three patients rejected for personal reasons. Notably, one patient (case 9) experienced disease progression due to poor compliance and lack of standard treatment but still achieved CR after just one cycle of the P3-GemOx regimen. Another patient (case 5), initially received two cycles of COEPL regimen, did not achieve PR but later reached PR following three cycles of P3-GemOx regimen. We present the imaging findings of two representative patients: One patient (case 20) initially presented with systemic lymph node involvement and intramedullary lesion infiltration (Figures 3A,B). Refractory to prior P-GemOx combined with Chidamide therapy, the patient achieved remarkable regression of all lesions after receiving the P3-GemOx regimen at our institution, attaining complete response (CR) following two treatment cycles (Figures 3C,D). Another patient (case 22) initially presented with tumor involvement confined to the nasopharynx (Figures 3E,F), but exhibited suboptimal response to initial P-GemOx plus radiotherapy. After receiving two cycles of P3-GemOx therapy at our institution, the patient achieved complete response (CR) (Figures 3G,H). Both patients met the criteria for and subsequently underwent allogeneic hematopoietic stem cell transplantation (allo-HSCT) following completion of P3-GemOx therapy. To date, they have maintained favorable clinical status post-transplantation. In this group, both the 1-year progression-free survival (PFS) and overall survival (OS) rates were reported at 100%. Patients receiving this regimen exhibited a tendency for improved PFS and OS (Figure 4).

### Adverse events

All patients experienced treatment-related adverse events (AEs) (Table 3). The most prevalent grade 3 or 4 AEs included anemia [seven patients (64%) in the PP-GemOx group vs. eight patients (72%) in the P3-GemOx group], neutropenia [nine patients (82%) in the PP-GemOx group vs. seven patients (64%) in the P3-GemOx group], and thrombocytopenia [six patients (54%) in the PP-GemOx group vs. seven patients (64%) in the P3-GemOx group]. There was no significant difference in the duration of neutropenia and thrombocytopenia between the two treatment groups (data not shown). One patient (case 2) developed grade 2 arrhythmia. Two patients receiving the PP-GemOx regimen (cases 11 and 17) ultimately died due to infection, while no patients died from bleeding. Besides, no other patients discontinued treatment due to treatment-related AEs, which were all manageable and resolved successfully.

**Table 3:** Adverse events related to the immunochemotherapy regimen.

Events	*n*/11 (%)
Adverse events related to PP-GemOx (*n* = 11)	
Grade 1–2	
Emesis/nausea	7/11 (64%)
Diarrhea	4/11 (36%)
Anemia	3/11 (27%)
Neutropenia	2/11 (18%)
Thrombocytopenia	3/11 (27%)
Transaminase	6/11 (54%)
Grade 3	
Anemia	4/11 (36%)
Neutropenia	5/11 (45%)
Thrombocytopenia	3/11 (27%)
Transaminase	3/11 (27%)
Grade 4	
Anemia	3/11 (27%)
Neutropenia	4/11 (36%)
Thrombocytopenia	3/11 (27%)
Adverse events related to P3-GemOx (*n* = 11)	
Grade 1–2	
Emesis/nausea	4/11 (36%)
Diarrhea	2/11 (18%)
Arrhythmia	1/11 (18%)
Neutropenia	2/11 (18%)
Thrombocytopenia	2/11 (18%)
Transaminase	5/11 (45%)
Grade 3	
Anemia	4/11 (36%)
Neutropenia	2/11 (18%)
Thrombocytopenia	2/11 (18%)
Transaminase	2/11 (18%)
Grade 4	
Anemia	4/11 (36%)
Neutropenia	5/11 (45%)
Thrombocytopenia	5/11 (45%)

## Discussion

This investigation constitutes the inaugural assessment of mitoxantrone liposomes (Plm60) integrated with PD-1 inhibitor-based chemotherapy in natural killer/T-cell lymphoma (NKTL), employing polyethylene glycol-modified formulations. Our data propose that strategic replacement of day 8 gemcitabine with PLM60 in the PP-GemOx protocol may constitute a viable therapeutic approach for advanced NKTL, demonstrating both safety and clinical efficacy. Longitudinal monitoring of circulating EBV DNA levels demonstrated significant correlations with therapeutic responses, establishing this biomarker as a valuable predictive tool.

Beyond its direct anti-neoplastic effects, the PP-GemOx regimen exhibits multifaceted immunomodulatory properties. Gemcitabine component demonstrates capacity to diminish myeloid-derived suppressor cell (MDSC) populations while polarizing tumor-associated macrophages toward immunostimulatory phenotypes (35–37). Concurrently, it enhances tumor antigen presentation through upregulation of MHC class I surface expression (38). Oxaliplatin contributes to immune activation by modulating lymphocyte ratios (CD8 + CTLs vs. Tregs) and potentiating innate immune cell functions, thereby promoting immunogenic cell death mechanisms (39, 40). PD-1 blockade synergistically amplifies these immune-mediated anti-tumor effects (41).

Therapeutic integration of mitoxantrone liposomes with PP-GemOx (P3-GemOx) appears to generate enhanced and sustained tumor control. Clinical validation emerged through universal treatment response (100% ORR) in our nine-patient cohort, comprising seven complete remissions (CR) and two partial responses (PR).

A key therapeutic advantage lies in facilitating CR attainment for subsequent hematopoietic stem cell transplantation (HSCT) consolidation. While role of HSCT in NKTL management remains controversial, current evidence identifies it as the most reliable modality for survival prolongation (42, 43). Analysis of 53 advanced-stage patients revealed differential relapse patterns: 60% (3/5) non-transplanted CR patients recurred within 24 months versus 16% (4/25) post-auto-HSCT cases, with durable remission observed in all patients maintaining CR > 12 months post-transplant (44). Retrospective evaluation of five refractory NKTL patients demonstrated sustained CR following HSCT (median follow-up 1911 days) (45). CIBMTR data from 25 advanced patients showed 2-year PFS/OS rates of 20%/24%, with pre-HSCT CR status significantly predicting survival (*p* < 0.001) (46).

The P3-GemOx regimen demonstrated a significantly higher objective response rate compared to the PP-GemOx regimen [100% (9 CR, 2 PR) vs. 63.6% (2 CR, 5 PR), *p* = 0.041, Fisher’s exact test]. Six P3-GemOx responders successfully underwent HSCT with favorable outcomes (three declinations for non-medical reasons), suggesting enhanced long-term prognosis potential. Our novel protocol demonstrates a notable advantage over conventional approaches, with the potential to optimize hematopoietic stem cell transplantation eligibility criteria and survival metrics (Table 4). Furthermore, to adjust for bias introduced by HSCT, we performed a 6-month landmark analysis. Only patients who survived beyond 6 months from the initiation of therapy were included, resulting in a cohort of 20 patients (11 in the P3-GemOx group and nine in the PP-GemOx group). Starting follow-up from the 6-month time point, the P3-GemOx group continued to demonstrate superior long-term survival (*p* = 0.014), indicating that the overall survival benefit associated with the P3-GemOx regimen observed in our study was not entirely driven by HSCT.

**Table 4:** Outcome of patients with advanced NK/T-cell lymphomas.

Regimens	Stage	ORR (%)	CR(%)	PFS	OS	References
SMILE	III/IV	Not reported	54	4-year: 60%	5-year: 47%	(47)
P-GemOx	III/IV	81	52	3-year: 24%	3-year: 58%	(48)
PP-GemOx	III/IV	88.9	77.8	1-year: 66.7%	1-year: 100%	(16)
P3-GemOx	III/IV	100	77.8	1-year: 100%	1-year: 100%	–

ORR, overall response rate; CR, complete remission; PFS, progression-free survival; OS, overall survival; SMILE, dexamethasone, methotrexate, ifosfamide, L-asparaginase, etoposide; P-GemOx, pegaspargase, gemcitabine, oxaliplatin; PP-GemOx, pegaspargase, gemcitabine, oxaliplatin, anti-programmed death 1 antibody; P3-GemOx, pegaspargase, gemcitabine, oxaliplatin, anti-programmed death 1 antibody, PLM60.

Safety analysis revealed that toxicities associated with the P3-GemOx regimen were manageable with supportive care, primarily manifesting as Grade 1–2 nausea/vomiting (36%) and transaminitis (45%), concurrently accompanied by Grade 3–4 cytopenias (anemia 73%, neutropenia 64%, thrombocytopenia 64%). Although the incidence of hematological toxicities was higher than the PP-GemOx baseline, intergroup comparisons using the Chi-square test or Fisher’s exact test indicated no significant difference in the occurrence of Grade 3–4 cytopenias (*p* > 0.05). Notably, no severe complications—such as pneumonia, renal impairment, or anaphylaxis—were observed, with only one case of Grade 2 arrhythmia reported. All adverse events were effectively managed through standard medical interventions, and no treatment discontinuations or fatal outcomes occurred due to AEs, suggesting that the toxicity profile of this regimen is clinically acceptable.

Study limitations warrant consideration: (1) The restricted sample size (*n* = 11 per group) not only impacts generalizability but also limits the statistical power for subgroup analyses and multivariable adjustment, potentially overestimating the effect size; (2) The undocumented immune-related adverse events (e.g., endocrine complications) mean the full safety profile of the combination, particularly long-term immune toxicities, remains incompletely characterized; (3) The preliminary nature of anti-tumor mechanism analyses precludes definitive conclusions regarding the specific contribution of mitoxantrone’s immunomodulatory effects versus its direct cytotoxicity. These limitations necessitate cautious interpretation of the high response rates and highlight the need for validation in a larger, more diverse patient population.

To explicitly address these limitations, we propose to conduct a prospective, multicenter, randomized controlled trial (RCT) focused on the P3-GemOx regimen in the future. This trial should include an expanded sample size sufficient to achieve statistical power for detecting clinically significant differences in primary endpoints such as progression-free survival (PFS) or overall survival (OS). Furthermore, correlative translational studies—such as dynamic profiling of peripheral immune cells and tumor microenvironment analyses before and after treatment—should be incorporated to elucidate the precise mechanisms of action.

In conclusion, P3-GemOx emerges as a promising therapeutic advancement for advanced NKTL, combining enhanced efficacy with acceptable tolerability. While clinical management of advanced NKTL has historically presented substantial challenges, this innovative regimen demonstrates potential to significantly improve patient outcomes through optimized treatment sequencing and response rates.

## Data Availability

The original contributions presented in the study are included in the article/Supplementary material, further inquiries can be directed to the corresponding author/s.
