# Supplementary material for: P3-GemOx as a novel immunochemotherapy candidate in NK/T-cell lymphoma management
Source: Front Med (Lausanne). 2025 Oct 23;12:1666601. doi: 10.3389/fmed.2025.1666601 (PMC12588983; doi:10.3389/fmed.2025.1666601)
Supplement: Supplementary file 1 [file Table_1.DOCX]

**Supplementary Table. Clinical characteristics of 22 patients with NKTL treated with the immunochemotherapy regimen**

| Case | Sex | Age, y | Sites | Marrow | Stage | ECOG score | PINK/PINK-E score | EBV DNA load |
| --- | --- | --- | --- | --- | --- | --- | --- | --- |
| 1 | M | 28 | skin of upper limbs | Positive | IV B | 1 | 3/4 | 1.16×10^4^ copies/ml |
| 2 | M | 57 | lymph nodes, liver | Positive | IV B | 1 | 3/4 | 2.31×10^4^ copies/ml |
| 3 | M | 27 | bone marrow | Positive | IV B | 1 | 2/3 | 9.23×10^5^ copies/ml |
| 4 | F | 40 | lymph nodes, liver | Positive | IV B | 1 | 3/4 | 1.28×10^5^ copies/ml |
| 5 | F | 36 | gingiva | Negative | IV A | 0 | 3/4 | <4×10^2^ copies/ml |
| 6 | M | 59 | nasopharynx | Negative | I_E_ A | 1 | 1/2 | <4×10^2^ copies/ml |
| 7 | M | 44 | nasopharynx | Positive | IV B | 1 | 3/4 | <4×10^2^ copies/ml |
| 8 | M | 41 | lymph nodes, liver, spleen | Positive | IV B | 1 | 3/4 | 3.64×10^4^ copies/ml |
| 9 | F | 37 | lymph nodes | Positive | IV B | 1 | 3/4 | <4×10^2^ copies/ml |
| 10 | F | 37 | skin of lower limbs | Negative | IV A | 1 | 3/4 | <4×10^2^ copies/ml |
| 11 | M | 55 | nasopharynx, pancreas | Negative | IV A | 1 | 3/4 | 2.87×10^3^ copies/ml |
| 12 | F | 51 | pancreas | Negative | IV B | 2 | 3/4 | 3.41×10^4^ copies/ml |
| 13 | M | 32 | bone marrow | Positive | IV B | 2 | 3/4 | 1.11×10^4^ copies/ml |
| 14 | M | 28 | lymph nodes | Negative | IV B | 1 | 3/4 | 2.5×10^3^ copies/ml |
| 15 | M | 46 | nasopharynx | Negative | II A | 1 | 0/1 | <4×10^2^ copies/ml |
| 16 | M | 21 | bone marrow | Positive | IV B | 1 | 3/4 | 6.6×10^2^ copies/ml |
| 17 | F | 18 | lymph nodes, bone | Positive | IV B | 1 | 3/4 | 3.59×10^4^ copies/ml |
| 18 | M | 76 | skin of lower limbs | Positive | IV B | 1 | 4/5 | 3.2×10^5^ copies/ml |
| 19 | F | 54 | transverse colon | Negative | II_E_ B | 1 | 1/2 | <4×10^2^ copies/ml |
| 20 | M | 28 | bone marrow | Positive | IV B | 1 | 3/4 | 1.86×10^4^ copies/ml |
| 21 | M | 14 | nasopharynx | Negative | I B | 1 | 1/2 | 2.78×10^3^ copies/ml |
| 22 | F | 56 | nasopharynx | Negative | I B | 1 | 1/2 | 9.83×10^3^ copies/ml |
